# Association of *ORAI1* Haplotypes with the Risk of HLA-B27 Positive Ankylosing Spondylitis

**DOI:** 10.1371/journal.pone.0020426

**Published:** 2011-06-01

**Authors:** James Cheng-Chung Wei, Jeng-Hsien Yen, Suh-Hang Hank Juo, Wei-Chiao Chen, Yu-Shiuan Wang, Yi-Ching Chiu, Tusty-Jiuan Hsieh, Yuh-Cherng Guo, Chun-Huang Huang, Ruey-Hong Wong, Hui-Po Wang, Ke-Li Tsai, Yang-Chang Wu, Hsueh-Wei Chang, Edward Hsi, Wei-Pin Chang, Wei-Chiao Chang

**Affiliations:** 1 Division of Allergy, Immunology and Rheumatology, Department of Medicine, Chung Shan Medical University Hospital, Institute of Medicine, Chung Shan Medical University, Graduate Institute of Integrated Medicine, China Medical University, Taichung, Taiwan; 2 Department of Medical Genetics, College of Medicine, Kaohsiung Medical University, Kaohsiung, Taiwan; 3 Cancer Center, Kaohsiung Medical University Hospital, Kaohsiung, Taiwan; 4 Division of Rheumatology, Department of Internal Medicine, Kaohsiung Medical University Hospital, Kaohsiung, Taiwan; 5 Department of Biomedical Science and Environmental Biology, Kaohsiung, Taiwan; 6 Graduate Institute of Medicine, Kaohsiung Medical University, Kaohsiung, Taiwan; 7 Department of Healthcare Management, Yuanpei University, HsinChu, Taiwan; 8 Department of Neurology, Kaohsiung Municipal Hsiao-Kang Hospital, Kaohsiung Medical University, Kaohsiung, Taiwan; 9 Department of Physiology, College of Medicine, Kaohsiung Medical University, Kaohsiung, Taiwan; 10 Department of Pharmacy, Taipei Medical University, Taipei, Taiwan; 11 Graduate Institute of Integrated Medicine, China Medical University, Taichung, Taiwan; University of Leuven, Rega Institute, Belgium

## Abstract

Ankylosing spondylitis (AS) is a chronic inflammation of the sacroiliac joints, spine and peripheral joints. The aetiology of ankylosing spondylitis is still unclear. Previous studies have indicated that genetics factors such as human leukocyte antigen *HLA-B27* associates to AS susceptibility. We carried out a case-control study to determine whether the genetic polymorphisms of *ORAI1* gene, a major component of store-operated calcium channels that involved the regulation of immune system, is a susceptibility factor to AS in a Taiwanese population. We enrolled 361 AS patients fulfilled the modified New York criteria and 379 controls from community. Five tagging single nucleotides polymorphisms (tSNPs) at *ORAI1* were selected from the data of Han Chinese population in HapMap project. Clinical statuses of AS were assessed by the Bath Ankylosing Spondylitis Disease Activity Index (BASDAI), Bath Ankylosing Spondylitis Functional Index (BASFI), and Bath Ankylosing Spondylitis Global Index (BAS-G). Our results indicated that subjects carrying the minor allele homozygote (CC) of the promoter SNP rs12313273 or TT homozygote of the SNP rs7135617 had an increased risk of HLA-B27 positive AS. The minor allele C of 3′UTR SNP rs712853 exerted a protective effect to HLA-B27 positive AS. Furthermore, the rs12313273/rs7135617 pairwise allele analysis found that C-G (OR 1.69, 95% CI 1.27, 2.25; p = 0.0003) and T-T (OR 1.75, 95% CI 1.36, 2.27; p<0.0001) haplotypes had a significantly association with the risk of HLA-B27-positive AS in comparison with the T-G carriers. This is the first study that indicate haplotypes of *ORAI1* (rs12313273 and rs7135617) are associated with the risk of HLA-B27 positive AS.

## Introduction

Ankylosing spondylitis (AS) is a systemic autoimmune disease affecting axial skeletons and peripheral joints[1]. AS ultimately limits the mobility of the spine and other joints, contributing to functional impairment[2]. Genetic factors have been strongly implicated in its pathogenesis. A twin study suggested that up to 97% of AS susceptibility was attributable to genetic factors[3]. AS was strongly associated with the human leukocyte antigen *HLA-B27* gene[4], but *HLA-B27* accounted for only 16% of the genetic load in AS[5]. HLA-B60, B61 and IL-1, IL-3R, and IL-23R complexes also have been proven to be important in the pathogenesis of AS[6]–[8]. A recent genome wide association study (GWAS) demonstrated immune related genes such as *ERAP1,* and *IL-23* as strong susceptibility genes to AS[9]. Consistently, immune related genes such as Toll-like receptor 4 and Toll-like receptor 5 are overexpressed in AS patients[10]. In addition, intergenic SNP rs10865331 was found to be susceptible to AS in the Spanish population[11]. *MSX2* genetic polymorphisms were associated with AS in Japanese but not Taiwanese[12]. In the Chinese Han population, Janus kinase 2 (JAK2) polymorphisms have been implicated to be involved in the susceptibility of AS[13]. Although several genes have been proposed to explain the susceptibility of AS, most genetic associations study cannot be replicated with other populations.

Ankylosing spondylitis (AS), an inflammatory disease, affects predominantly axial skeleton and sacroiliac joints. Therefore, molecules involved in the regulation of calcification, autoimmune and/or inflammation are good candidates for the AS susceptibility genes. Calcium-dependent pathways control diverse physiological functions including enzyme metabolism, immune responses and inflammatory activation[14]. In non-excitable cells such as T cells and B cells, Ca^2+^ entry was mainly through store-operated calcium channels to control immunological reactions[15]. Orai1 (also called CRACM1) consisted four transmembrane domains and functioned as a pore forming subunit of store-operated calcium channels[16]. Orai1 protein was highly expressed in bone tissues. Functional studies in *CRACM1* deficient mice indicated the dysfunction of mast cells, and attenuation of cytokine release (TNF-α and IL-6)[17]. *ORAI1*-R91W mutations disrupt the function of store-operated calcium channels resulting in the lack of Ca^2+^ influx, defective T cell activation, and immunodeficiency[18]. Additionally, there were lines of evidence for a role of store-operated calcium channel in the modulation of transcription factors including NFκB and NFAT. A great number of NFκB- and/or NFAT-mediated genes were critical for maintaining the immune system[19].

In this study, we examined the association between the *ORAI1* polymorphisms and the risk for AS using a case-control study. The relationship between AS activity index (BASDAI, BASFI, BAS-G), and genetic polymorphisms of *ORAI1* was also evaluated.

## Materials and Methods

### Study Subjects

Patients were solicited sequentially at Chung Shan Medical University Hospital in Taichung, Taiwan. AS patients who met selection criteria were asked to participate in the study. Informed consent was obtained before any data was collected from the respondents. Three selection criteria were used to recruit subjects: (1) AS diagnosis by the modified New York criteria[20]; (2) fluent Chinese language speakers; and (3) no obvious cognitive impairment. Sacroiliitis was confirmed by a qualified radiologist or rheumatologists, and AS diagnosis was confirmed by a qualified rheumatologist. A total of 361 unrelated AS patients were included in the study as cases. A detailed clinical history was recorded by the physician at enrollment. 29 (8%) AS patients whose age are less than 18. The mean duration of the symptoms was 7.5 years. 100% of AS patients in this study have sacroiliitis. A total of 379 control subjects were recruited from the general population who volunteered to participate in our study while receiving a health screening examination at the Kaohsiung Medical University Hospital. All the subjects gave the consent form. The study protocol conformed to the Declaration of Helsinki and study was approved by the Institute Review Board of each Hospital.

### Clinical Evaluations

Disease activity and functional status were assessed by the Chinese versions of the BASDAI, the BASFI, and the Bath Ankylosing Spondylitis Global (BAS-G) Score. Good reliability (0.87 to 0.94) and validity (0.92 to 0.94) of these Chinese methods have been documented[21].

### Laboratory Analyses

Venous blood was collected during medical surveillance, stored at 4°C, and processed on the same day. The blood was centrifuged to separate the serum and the cells. All specimens were stored under −70°C until analysis. HLA-B27 carriage had previously been assessed by flow cytometry[22]. Genotyping is performed using TaqMan PCR. Briefly, Taqman probes are labeled with different fluorescent markers. PCR primers and TaqMan probes are designed with SNP sites. Reactions are performed in 96 well microplates with ABI 7500 thermal cycles (Applied Biosystems, Foster City, USA). Fluorescence is measured by the ABI Real Time PCR system. By reading the fluorescence from PCR product, possible genotypes can be identified. Results are analyzed with the ABI SDS software version 1.2.3.

### Statistical Analysis

Genotype distributions of the five tagging single nucleotide polymorphisms (tSNPs) were tested for Hardy-Weinberg equilibrium (HWE), which means the allelic distribution between all populations and our study was not different (*P*>0.05). Chi-squared test was used to compare the genotypes distribution or allele frequencies between AS patients and controls. Analysis of variance (ANOVA) was used to compare the mean of continuous variables (BASDAI, BASFI, and BAS-G) among different genotypes in AS patients. Multiple regression analysis was used to adjust for age and sex. A *p* value after the Bonferroni correction less than 0.01 is considered significant. The analyses were performed by using SAS 9.1 statistical software. Linkage disequilibrium (LD) was assessed for any pair of SNPs and haplotype blocks were defined using the default setting of the Haploview software[23].

## Results

### Basic and Clinical Characteristics of the Subjects

**Figure 1:**
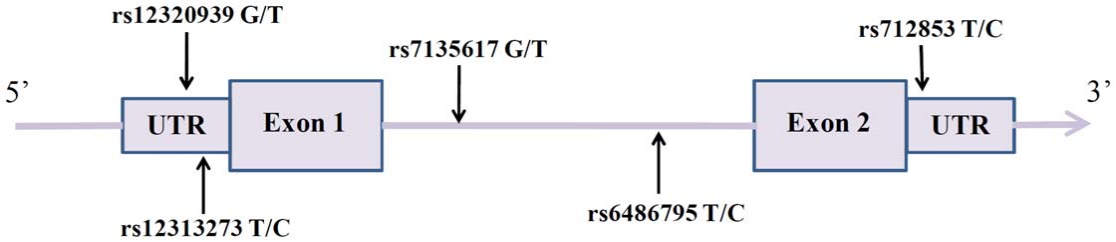
A graphical overview of genotyped polymorphisms identified in relation to the exon/intron structure of the human *ORAI1* gene.

In this study, we selected five tSNPs of *ORAI1* (rs12313273, rs6486795, rs7135617, rs12320939, and rs712853) with minor allele frequency >5% from the Han Chinese in Beijing (CHB) population in the HapMap database (http://www.hapmap.org). A graphical overview of genotyped polymorphisms was shown in Figure 1. Two polymorphisms (rs12313273, rs12320939) of *ORAI1* located in the promoter area, while two polymorphisms (rs6486795, rs7135617) in the intron and one (rs712853) in the 3′ untranslated region (UTR). A total of 361 AS patients and 379 controls were recruited in this study. Table 1 showed the characteristics of the subjects. The mean age (years) and standard deviation (S.D.) were 33.5±12.8 for cases and 28.3±15.2 for controls. More than 67.9% of cases and 69.9% of controls were male. 87.3% (315/361) AS subjects were HLA-B27 positive and their mean BASDAI, mean BASFI, and mean BAS-G scores were 4.1±2.3, 1.9±2.2, and 4.3±2.8, respectively.

**Table 1:** Basal characteristics and clinical features of patients with ankylosing spondylitis (AS) and of normal controls.

Characteristics	Patients with AS	Normal Controls
Number of subjects	361	379
Gender:male, No (%)	245 (67.9%)	265 (69.9%)^a^
Age (years)^b^	33.5±12.8	28.3±15.2^c^
Range	6–69	18–80
HLA-B27(+)	315 (87.3%)	
BASDAI (0–10)	4.1±2.3	
BASFI (0–10)	1.9±2.2	
BAS-G (0–10)	4.3±2.8	

^***a***^ *P* = 0.546 ∘.

^***b***^means ± S.D.

^***c***^ *P*<0.0001.

### Association of *ORAI1* genetic polymorphisms for the susceptibility of HLA-B27-positive or negative patients with AS

The genotypic frequencies of SNPs among the study subjects were shown in Table 2, and the distribution of genotypes was in HWE in controls. Two SNPs, rs7135617 and rs712853, showed significant associations with HLA-B27-positive AS patients in the recessive model. The C allele of rs712853 was associated with a lower AS risk (OR: 0.46, *P* = 0.002) than the rs712853 T allele. The adjustment for age and sex did not change the association for rs12313273 or rs712853 in the recessive model or genotype model. The association for rs7135617 or rs712853 was still significant even after the Bonferroni correction (*P*<0.01). In addition, a borderline significant association between the genotypes and allele frequency of rs12313273 in control subjects and HLA-B27 positive patients was obtained with a *P* value = 0.012 under the recessive model. Individuals with the rs12313273 homozygous C/C genotype had a 2.11-fold increased risk of AS compared with those with the C/T and T/T genotypes.

**Table 2:** Genotyping and allele frequency of *ORAI1* tSNP in HLA-B27(+) or HLA-B27(-) with ankylosing spondylitis (AS) and controls.

	Genotype	HLA-B27(+) (n = 315) (%)	HLA-B27(-) (n = 46) (%)	Control Subjects (n = 379) (%)	Recessive Odds ratio†	Recessive *P* Value†	Recessive Odds ratio‡	Recessive *P* Value‡
rs12320939	TT	68 (22.2)	15 (32.6)	90 (26.1)	0.89	0.511	1.68	0.138
	GT	142 (46.4)	22 (47.8)	165 (47.8)				
	GG	96 (31.4)	9 (19.6)	90 (26.1)				
rs12313273	CC	34 (10.9)	7 (15.6)	20 (5.7)	2.11	0.012	3.32	0.015
	CT	116 (37.3)	18 (40.0)	148 (41.8)				
	TT	161 (51.8)	20 (44.4)	186 (52.5)				
rs7135617	TT	72 (23.2)	6 (13.7)	60 (17.0)	1.70	**0.008** *	0.90	0.817
	GT	139 (44.9)	21 (47.7)	164 (46.6)				
	GG	99 (31.9)	17 (38.6)	128 (36.4)				
rs6486795	CC	50 (16.1)	10 (22.2)	54 (15.6)	1.13	0.581	1.91	0.108
	CT	126 (40.7)	21 (46.7)	159 (45.8)				
	TT	134 (43.2)	14 (31.1)	134 (38.6)				
rs712853	CC	25 (8.1)	1 (2.2)	59 (16.2)	0.46	**0.002** *	0.13	0.045
	CT	109 (35.3)	24 (52.2)	153 (42.0)				
	TT	175 (56.6)	21 (45.6)	152 (41.8)				

*Significant (*P<0.01*) values are in bold.

†Adjusted the effects of age and sex for HLA-B27 positive patients compared with controls.

‡Adjusted the effects of age and sex for HLA-B27 negative patients compared with controls.

### No Association of *ORAI1* genetic polymorphisms with the disease activity of AS

We further analyzed the relationship between disease activity (BASDAI, BASFI and BAS-G) and the five polymorphisms of *ORAI1* among AS patients. BASFI is strongly affected by disease duration, therefore, adjustment for disease duration and BASFI was performed. However, none of SNPs reached a nominal significant level of 0.05 (Table 3). After adjustment for the effects of age and sex, the polymorphisms of *ORAI1* still failed to show any significant association with the severity of AS (Table 3). Subset analysis on cases with HLA-B27 positive or negative did not yield any significant results (data not shown).

**Table 3:** Difference in the scores of BASDAI, BASFI, and BAS-G among AS patients stratified by different *ORAI1* alleles.

SNP	Alleles	BASDAI	BASFI	BAS-G
rs12320939	T/T	4.1±2.4^a^	1.7±2.1	4.1±2.9
	G/T	4.1±2.3	1.9±2.1	4.2±2.8
	G/G	4.3±2.3	2.1±2.4	4.5±2.8
Unadjusted *P*-value		0.86	0.52	0.57
Adjusted *P*-value		0.86†	0.51§	0.57†
rs12313273	C/C	3.2±2.3	1.3±2.1	3.3±2.8
	C/T	4.4±2.4	1.9±2.1	4.4±2.8
	T/T	4.2±2.2	2.1±2.3	4.4±2.9
Unadjusted *P*-value		0.02	0.14	0.10
Adjusted *P*-value		0.02†	0.12§	0.09†
rs7135617	T/T	4.3±2.3	2.3±2.5	4.7±2.9
	G/T	4.1±2.3	2.0±2.2	4.1±2.8
	G/G	4.1±2.4	1.6±1.9	4.2±2.8
Unadjusted *P*-value		0.72	0.12	0.46
Adjusted *P*-value		0.72†	0.09§	0.46†
rs6486795	C/C	3.7±2.3	1.5±2.0	3.5±2.7
	C/T	4.2±2.3	1.9±2.1	4.2±2.7
	T/T	4.2±2.2	2.2±2.4	4.6±2.9
Unadjusted *P*-value		0.40	0.19	0.10
Adjusted *P*-value		0.40†	0.17§	0.10†
rs712853	C/C	3.9±1.9	2.3±2.0	4.3±2.7
	C/T	4.2±2.1	1.8±2.1	4.2±2.8
	T/T	4.1±2.5	2.0±2.3	4.3±2.9
Unadjusted *P*-value		0.88	0.64	0.95
Adjusted *P*-value		0.88†	0.61§	0.95†

a Data represent means ± S.D.

†Adjusted the effects of age and sex.

§Adjusted the effects of age, sex and disease duration.

### Haplotype Analysis of *ORAI1* genetic polymorphisms in the susceptibility of HLA-B27-positive or negative patients with AS

**Figure 2:**
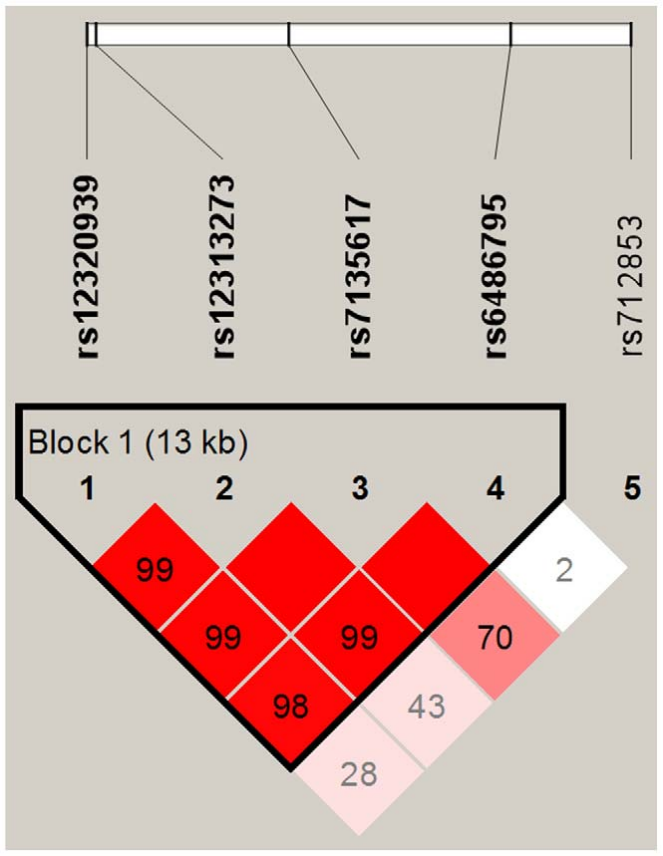
*ORAI1* gene LD and haplotype block structure in AS. The number on the cell is the LOD score of D'.

We calculated pairwise linkage disequilibrium (LD) (Figure 2) and analyzed three common haplotypes using the Haploview 4.2 program and PHASE version 2.1, respectively. The haplotype frequency of rs12313273/rs7135617 among the study subjects was shown in Table 4. rs12313273/rs7135617 pairwise allele analysis indicated that C–G (AOR  = 1.69; 95% CI, 1.27–2.25; *P* = 0.0003), and T-T (AOR  = 1.75; 95% CI, 1.36–2.27; *P*<0.0001) had a significant association with the risk of HLA-B27-positive AS in comparison with the T-G haplotype under a recessive model. However, none of haplotypes was significantly associated with HLA-B27-negative AS patients (Table 4).

**Table 4:** Haplotype frequency of the *ORAI1* gene in HLA-B27(+) or HLA-B27(-) patients with ankylosing spondylitis and controls.

rs12313273/rs7135617	HLA-B27(+) (n = 315) (%)	HLA-B27(-) (n = 46) (%)	Control Subjects (n = 379) (%)	AOR (95% CI)†	*P* Value†	AOR (95% CI)‡	*P* Value‡
C/G	184 (30.0)	30 (33.3)	186 (26.8)	1.69 (1.27–2.25)	**0.0003** *	1.65 (0.95–2.87)	0.078
T/T	280 (45.6)	34 (37.8)	282 (40.6)	1.75 (1.36–2.27)	**<0.0001** *	1.25 (0.73–2.13)	0.413
T/G	150 (24.4)	26 (28.9)	226 (32.6)	Reference		Reference	

*Significant (*P<0.05*) values are in bold.

†Adjusted the effects of age and sex for HLA-B27 positive patients compared with controls.

‡Adjusted the effects of age and sex for HLA-B27 negative patients compared with controls.

### No Association between *ORAI1* Haplotypes and the disease activity of AS

We further analyzed the relationship between disease activity and rs12313273/rs7135617 haplotypes among AS patients. As shown in table 5, none of the rs12313273/rs7135617 pairwise allele analysis tested in this study showed a significant association between AS and BASDAI, BASFI, and BAS-G. After adjustment for the effects of age, gender and disease duration, the haplotype analysis of rs12313273/rs7135617 still failed to show any significant results with the disease activity of AS.

**Table 5:** Difference in the scores of BASDAI, BASFI, and BAS-G among AS patients stratified by different *ORAI1* haplotypes.

rs12313273/rs7135617	BASDAI	BASFI	BAS-G
C/G	4.0±2.4^a^	1.7±2.1	4.0±2.8
T/T	4.2±2.3	2.1±2.3	4.4±2.8
T/G	4.2±2.2	1.9±2.0	4.4±2.8
Unadjusted P-value	0.46	0.07	0.27
Adjusted P-value	0.46†	0.03§	0.26†

a Data represent mean ± S.D.

†Adjusted the effects of age and sex.

§Adjusted the effects of age, sex and disease duration.

## Discussion

Our results first revealed that genetic polymorphisms of *ORAI1* rs12313273 (located in the promoter), rs7135617 (located in the intron) and rs712853 (located in the 3′UTR) were associated with susceptibility to AS in a Taiwanese population. Results from pairwise allele analysis for rs12313273/rs7135617 indicated that C-G and T-T haplotypes associated with a significantly higher risk of HLA-B27 positive AS in comparison with the T-G haplotypes. Several lines of evidence indicated the importance of SNPs at the promoter and 3′UTR in gene expression. Studies showed that a promoter SNP of the tumor-necrosis factor α (*TNF-α*) was associated with the development of AS via attenuation of *TNF-α* gene expression [24]. Furthermore, SNPs at the 3′UTR may interfere with mRNA stability or protein translation by interacting with microRNAs[25]. MicroRNAs have been shown to play a critical role in the regulation of gene expression in immune diseases, including psoriatic arthritis susceptibility[26]. A recent study demonstrated that a 3′UTR polymorphisms of IL-1R associated kinase (*IRAK1*) gene was associated with rheumatoid arthritis (RA) susceptibility[26]. The RA-associated SNP of *IRAK1* was in the binding site of microRNA-146a[26]. Results obtained in *IRAK1* implied that 3′UTR polymorphism at *ORAI1* might affect susceptibility to AS via similar mechanisms (microRNA-relevant expression alteration).

Despite the strong association of *HLA-B27* gene with AS, other genes within and/or outside the MHC were reported to involve in the susceptibility of AS[6], [8], [9]. Wei et al[8]. found that *HLA-B60* and *HLA-B61* genes were associated with AS development among *HLA-B27* negative patients which might be due to the similar T-cell epitopes of HLA-B60 and HLA-B27 [27]. In vivo, the inflammation in the joint destruction was regulated by IL-1α and IL-1β, and IL-1 receptor antagonist (IL-1Ra) combined with IL-1 receptor to inhibit the IL-1[28]. In addition, the genetic variants in the IL-1F10.3, IL-1RN.4, IL-1RN.VNTR, IL-1RN6/1 and IL-1RN6/2 were significantly associated with occurrence of AS [29]. Shiau et al., observed that the distribution of TNF-α G-238A genotypes and alleles, as well as that of G-308A genotypes and alleles, between AS patients and controls were significantly different[30]. Both IL-1β and TNF-α pathways coordinated multiple signaling events leading to the increase of intracellular calcium concentration [31]. Association study in Chinese Han population revealed that JAK2 played an important role in the susceptibility to AS [13]. The increase of cytosolic calcium resulted in the activation of JAK-STAT pathways, which, in turn, involved in the regulation of proinflammatory genes [32]. Our association studies in AS provided some indirect evidence that supported a role of calcium signaling in the susceptibility to AS. Therefore, to understand the functional role of *ORAI1* polymorphisms in the regulation of JAK2/STAT pathways may gain more insight to the disease pathogenesis.

Calcium influx through store-operated calcium channels has been shown its importance in a variety of diseases such as hypertension, arterial injury and severe combined immunodeficiency disease (SCID)[33]. A point mutation of *ORAI1* that resulted in the reduction of store-operated Ca^2+^ influx and cytokine release was found in the SCID patients[18]. The biophysical characteristics of store-operated calcium channel can be altered by the changed composition of heteromeric *ORAI* channels. The Ca^2+^ selectivity of homomeric *ORAI1* or *ORAI3* was higher than that of heteromeric *ORAI1* and *ORAI3* [34]. Additionally, knockdown of *ORAI1* can inhibit cell proliferation via attenuation of store-operated Ca^2+^ influx [35], [36]. Importantly, overexpression of *ORAI1* may influence the successful coupling between *ORAI* subunits or lose sensitivity to the store-depletion signals that lead to the dysfunction of store-operated calcium channel [37], [38]. The expression level of *ORAI* subunits hence could significantly contribute to the intracellular Ca^2+^ mobilization and physiological functions. The mechanism of how *ORAI1* gene being regulated is still unclear. Our results indicated that genetic polymorphisms of *ORAI1* (rs12313273 and rs712853) were associated with the risk of AS. With these findings, our study may offer a clue to better understand the regulation mechanism of *ORAI1*.

In conclusion, our research was the first study to pinpoint the association between genetic polymorphisms of *ORAI1* and the risk of AS. Our study indicated that haplotypes of rs12313273/rs7135617 had a significantly association with the risk of HLA-B27-positive AS but none of them was associated with theHLA-B27-negatve AS. We attribute this to the case number (46 HLA-B27negative AS patients), due to a small statistical power. Another possible explanation, that should be mentioned, is that these haplotypes are associated with the HLA- B27 per se and not exactly to AS.

We also analyzed the relationship between age of onset, hip involvement, erythrocyte sedimentation rate (ESR), C reactive protein (CRP) and *ORAI1* genotypes. However, no statistically significant association between genotypes and phenotypes were found (data not shown).We acknowledged that the sample size in the study was under-powered to detect the small genetic effect of *ORAI1* in the disease activity such as BASDAI/ BASFI, BAS-G., ESR or CRP. These findings need to be replicated in another population with a larger sample size.
